# Solid-state AIEnh-circularly polarised luminescence of chiral perylene diimide fluorophores†

**DOI:** 10.1039/c8ra09785b

**Published:** 2019-01-14

**Authors:** Ayano Taniguchi, Daiki Kaji, Nobuyuki Hara, Ryosuke Murata, Shogo Akiyama, Takunori Harada, Atsushi Sudo, Hiroyuki Nishikawa, Yoshitane Imai

**Affiliations:** Department of Applied Chemistry, Faculty of Science and Engineering, Kindai University 3-4-1 Kowakae Higashi-Osaka Osaka 577-8502 Japan y-imai@apch.kindai.ac.jp; Graduate School of Science and Engineering, Ibaraki University 2-1-1 Bunkyo Mito Ibaraki 310-8512 Japan hiroyuki.nishikawa.sci@vc.ibaraki.ac.jp; Department of Integrated Science and Technology, Faculty of Science and Technology, Oita University Dannoharu 700 Oita City 870-1192 Japan

## Abstract

Solid-state organic fluorescent materials are important for the development of electroluminescent sensing devices. Herein, we report that *N*,*N′*-bis((*R*)-1-phenylethyl)perylene-3,4,9,10-tetracarboxylic diimide [(*R*,*R*)-BPP] and its antipode [(*S*,*S*)-BPP], which contain extended π-electrons through planar perylenes, emit solid-state aggregation-induced-enhanced (AIEnh) circularly polarised luminescence (CPL) in inorganic (KBr) pellets and organic-polymer-film (PMMA- and *myo*-IPU-film) states; this CPL is difficult to observe in solution. These chiral perylene fluorophores emit AIEnh-CPL with high dissymmetry factors (*g*_CPL_) (up to 2.4 × 10^−3^) and high quantum yields (*Φ*_F_, up to 0.43) in the three solid matrices.

## Abstract 2

Chiral *N*,*N*′-bis(1-phenylethyl)perylene-3,4,9,10-tetracarboxylic acid diimide (BPP) exhibits solid-state aggregation-induced enhanced circularly polarized luminescence (AIEnh-CPL) in KBr, PMMA, and myo-IPU matrices..

## Graphical abstract

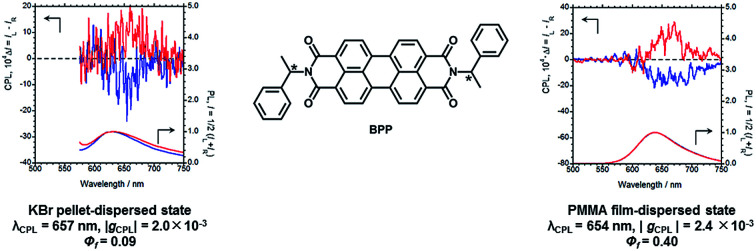

## Introduction

Because of their potential applications to electroluminescence (EL) and optical-sensing devices, solid-state organic fluorescent materials have attracted much attention.^1^ In particular, optically active organic and inorganic luminophores that emit circularly polarised luminescence (CPL) with high dissymmetry factors (*g*_CPL_) and high quantum yields (*Φ*_F_) have received significant attention in the field of circularly polarised organic light-emitting diodes (CP-OLEDs).^2^ Recently, aggregation-induced-enhanced (AIEnh) CPL has also attracted much interest. However, AIEnh-CPL is almost always emitted from highly concentrated solutions.^3^ Therefore, systems that emit solid-state AIEnh-CPL are highly desired. With the aim of providing a common design approach to novel chiral solid-state AIEnh organic fluorophores, in this study we prepared solid-state AIEnh circularly polarised luminophores by doping chiral *N*,*N*′-bis((*R*)-1-phenylethyl)perylene-3,4,9,10-tetracarboxylic diimide [(*R*,*R*)-BPP] and its antipode [(*S*,*S*)-BPP] into various types of solid matrix. The two BPPs are chiral bi-substituted perylene fluorophores composed of extended π-electron planar perylenes. We then examined the AIEnh-CPL emissions from these imbedded BPP systems as AIEnh-fluorophore models.^4^ KBr was used as the solid inorganic matrix, while poly(methyl methacrylate) (PMMA) and polyurethane (*myo*-IPU) were chosen as solid organic polymer matrices.^5^*Myo*-IPU is derived from naturally occurring *myo*-inositol (a *meso*-hexose) and characteristically has a higher glass transition temperature (*T*_g_ ∼150 °C) than conventional PMMA (*T*_g_ ∼105 °C).

**Fig. 1:**
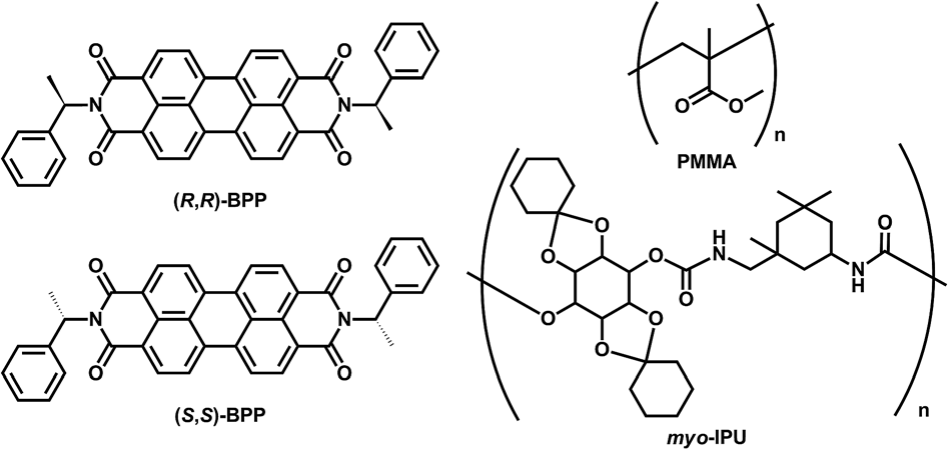
The chiral perylene fluorophores ((*R*,*R*)-BPP and (*S*,*S*)-BPP) and organic polymer matrices (PMMA and *myo*-IPU) used in this study.

Herein, we report that (*R*,*R*)- and (*S*,*S*)-BPP emit solid-state AIEnh-CPL with high quantum efficiencies when imbedded in solid inorganic and organic polymer matrices (Fig. 1).

## Experimental

### General

The KBr and PMMA matrices for solid pellets and films were purchased from JASCO (Tokyo, Japan) and the FUJIFILM Wako Pure Chemical Corp. (Osaka, Japan), respectively. Polyurethane (*myo*-IPU) was prepared by a previously reported method.^5^ Chloroform (CHCl_3_) used for the determination of solution-state optical properties was purchased from the FUJIFILM Wako Pure Chemical Corp. (Osaka, Japan).

### Syntheses of *N*,*N*′-bis((*R*)-1-phenylethyl)perylene-3,4,9,10-tetracarboxylic diimide [(*R*,*R*)-BPP] and (*S*,*S*)-BPP

(*R*,*R*)-BPP and (*S*,*S*)-BPP were prepared with reference to a previously reported method.^4^ A mixture of 3,4,9,10-perylenetetracarboxylic dianhydride (430.0 mg, 1.1 mmol), (*R*)-1-phenylethylamine (0.3 mL, 2.4 mmol), and zinc acetate dihydrate (240.1 mg, 1.1 mmol) in quinolone (20 mL) was heated with stirring at 180 °C under nitrogen for 4 h. After cooling to room temperature, the reaction mixture was treated with 2 N HCl and stirred at room temperature overnight. The resulting dark-red precipitate was collected by filtration, washed thoroughly with water and methanol (MeOH), and dried *in vacuo*. The crude product was purified by silica-gel column chromatography using CHCl_3_/EtOAc (20 : 1, v/v) as the eluent, to give 523.2 mg (79% yield) of (*R*,*R*)-BPP as a deep-red solid. The *S*-enantiomer, namely (*S*,*S*)-BPP, was synthesised from (*S*)-1-phenylethylamine by the same procedure to provide a deep-red solid (76% yield): ^1^H NMR for (*R*,*R*)-BPP (CDCl_3_ 500 MHz) *δ* 2.06 (d, *J* = 7.5 Hz, 3H), 6.60 (q, *J* = 7.5 Hz, 2H), 7.29 (t, *J* = 7.5 Hz, 2H), 7.38 (t, *J* = 7.5 Hz, 4H), 7.57 (d, *J* = 7.5 Hz, 4H), 8.58 (d, *J* = 8.0 Hz, 4H), 8.66 (d, *J* = 8.0 Hz, 4H); ^13^C NMR (CDCl_3_ 125 MHz) *δ* 16.3, 50.5, 122.9, 123.5, 126.1, 127.2, 127.5, 128.2, 129.2, 131.3, 134.3, 140.5, 163.3.

### Unpolarised photoluminescence (PL) quantum yields

Samples were dispersed in KBr pellets in the following way. Microcrystalline powdered samples (10 μg) were finely ground with 120 mg of KBr, and the mixed powder was compressed at 80 MPa cm^−2^ under vacuum for 25 min to provide a 10 mm-diameter, 0.7 mm-thick transparent disk. Samples dispersed in PMMA or *myo*-IPU films were prepared using PMMA or *myo*-IPU (1.0 × 10^−2^ M) in a spin coater at 3000 rpm (Opticoat MS-A100, Mikasa, Tokyo, Japan).

Absolute unpolarised photoluminescence (PL) quantum yields (*Φ*_F_) of the KBr-pellet-dispersed states, the PMMA- or *myo*-IPU-film-dispersed states, and CHCl_3_ solutions were determined using a Hamamatsu Photonics C9920-02 absolute quantum yield spectrometer (Hamamatsu, Japan) in air at room temperature, at excitation wavelengths of 480 nm (KBr) and 450 nm (polymer film), respectively, and at 450 nm for the CHCl_3_ solution. A 10 mm path length was used for solution-phase spectroscopy.

### Solid-state PL and CPL spectroscopy

Solid-state PL and CPL spectra of the KBr-pelleted samples were acquired by comprehensive chiroptical spectrophotometry (CCS, JASCO, Tokyo, Japan).^6^ CPL spectra were recorded with a slit width of 33 mm and a 10 nm spectral bandwidth for the excitation and emission monochromators. The PL and CPL spectra of the PMMA or *myo*-IPU films and the CHCl_3_ solutions were acquired using a JASCO CPL-300 spectrofluoropolarimeter (Tokyo, Japan) at room temperature, at a scattering angle of 0° upon excitation with unpolarised, monochromated incident light with a 10 nm bandwidth. An excitation wavelength of 480 nm was used for the KBr-pelleted samples, while 450 nm was used for the PMMA or *myo*-IPU films, and for the CHCl_3_ solutions. A 10 mm path length was used for solution-phase spectroscopy.

### Circular dichroism (CD) and UV-Vis absorption spectroscopy

Circular dichroism (CD) and UV-Vis absorption spectra of the KBr-pelleted samples were acquired by CCS at room temperature.^6^ The CD and UV-Vis absorption spectra of PMMA- or *myo*-IPU-film-dispersed states and CHCl_3_ solutions were recorded with a JASCO J-820 spectropolarimeter at room temperature. The KBr-pelleted and PMMA (or *myo*-IPU) films were prepared as described for the samples used for solid-state PL and CPL spectroscopy. A 1 mm path length was used for solution-phase spectroscopy.

## Results and discussion

**Fig. 2:**
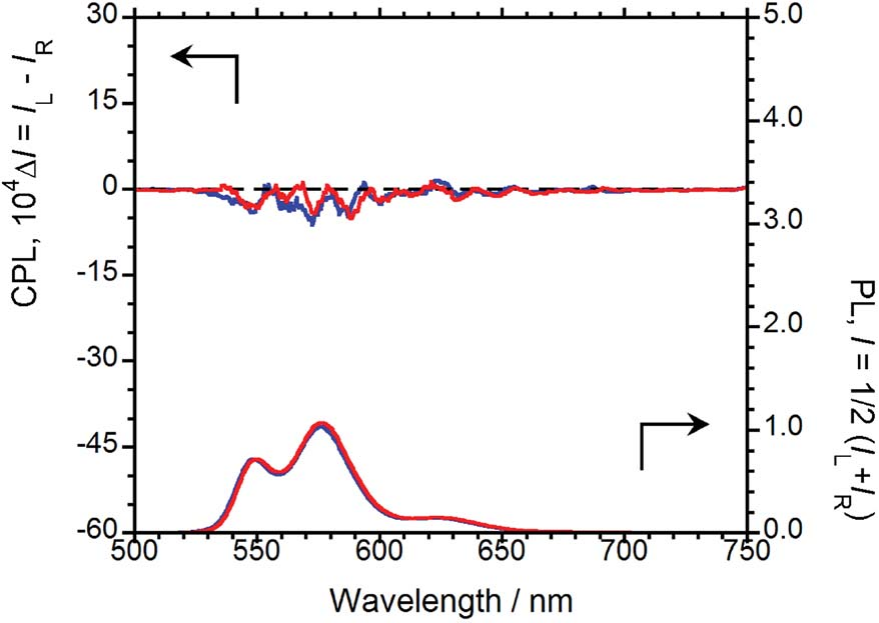
CPL (upper) and PL (lower) spectra of (*R*,*R*)-BPP (blue) and (*S*,*S*)-BPP (red) in CHCl_3_ (1.0 × 10^−4^ M).

Prior to elucidating the solid-state chiroptical properties of (*R*,*R*)-BPP and (*S*,*S*)-BPP, we first investigated their PL, CPL, and CD spectral characteristics in CHCl_3_ solution, at room temperature. To determine whether or not the obtained results are due to artefacts introduced by (*R*,*R*)-BPP and other factors, the CPL spectrum of (*S*,*S*)-BPP was also acquired. The CPL and PL spectra of (*R*,*R*)-BPP and (*S*,*S*)-BPP are shown in Fig. 2 ((*R*,*R*)-BPP in blue; (*S*,*S*)-BPP in red). When dissolved in CHCl_3_ (1.0 × 10^−4^ M), (*R*,*R*)-BPP exhibited PL at 549, 576, 623 nm with an absolute photoluminescence quantum yield (*Φ*_F_) of 0.76. Unfortunately, meaningful CPL spectra of chiral BPP could not be acquired under these conditions.

**Fig. 3:**
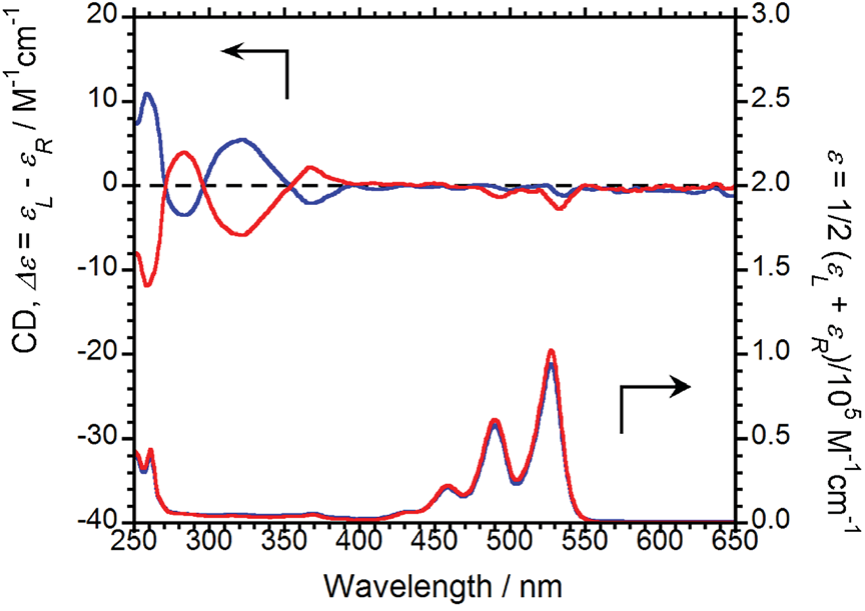
CD (upper) and UV-Vis absorption (lower) spectra of (*R*,*R*)-BPP (blue) and (*S*,*S*)-BPP (red) in CHCl_3_ (1.0 × 10^−4^ M).

The CD and UV-Vis absorption spectra of (*R*,*R*)-BPP and (*S*,*S*)-BPP in CHCl_3_ are shown in Fig. 3; as expected, the CD spectra are almost mirror images.

Absorption bands at 459, 490, and 527 nm that originate from π–π* transitions in the perylene units are commonly observed, irrespective of the chirality of the sample. As same as CPL properties in the solution-dissolved state, these corresponding CD bands could not be mainly observed.

**Fig. 4:**
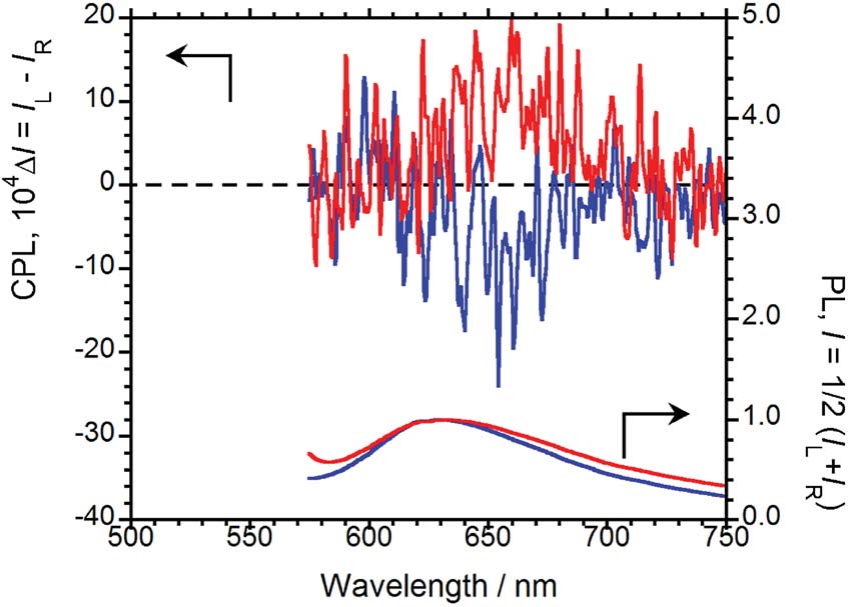
CPL (upper) and PL (lower) spectra of (*R*,*R*)-BPP (blue) and (*S*,*S*)-BPP (red) in KBr-pellet-dispersed states.

For the chiral BPPs to emit CPL, we turned to solid-state AIEnh-CPL spectroscopy. The solid-state PL and CPL spectra of (*R*,*R*)-BPP were acquired in the inorganic KBr matrix, the results of which are shown in Fig. 4. In contrast to the results in CHCl_3_ solution, solid-state CPL was observed from the KBr-pelleted sample; (*R*,*R*)-BPP in the KBr pellet exhibited a *λ*_CPL_ of 657 nm with a corresponding *Φ*_F_ of 0.09. When the spectrum of the KBr-pellet-dispersed state is compared with that of the CHCl_3_ solution, quite different *λ*_PL_ values are observed (576 nm for CHCl_3_ and 632 nm for KBr). The *λ*_PL_ of the KBr pellet suggests that CPL from this state is AIEnh CPL derived from the aggregation of (*R*,*R*)-BPP in the solid state. Unfortunately, the value of *Φ*_F_ in the KBr-dispersed state was smaller than that in CHCl_3_ solution, which is ascribable to fluorescence quenching under high-density conditions in the solid compared to the low-concentration conditions in solution. In addition, the external heavy-atom (Br) effect may also be partially responsible.

A negative (−) CPL spectrum was observed for (*R*,*R*)-BPP in the solid state. To determine whether or not these results are due to artefacts introduced by (*R*,*R*)-BPP and the KBr matrix, the CPL spectrum of (*S*,*S*)-BPP was acquired (Fig. 4). (*R*,*R*)-BPP and (*S*,*S*)-BPP in KBr matrices exhibit almost mirror-image CPL spectra. The magnitude of the circular polarisation in the excited state is defined as *g*_CPL_ = Δ*I*/*I* = (*I*_L_ − *I*_R_)/(*I*_L_ + *I*_R_), where *I*_L_ and *I*_R_ are the output signals for left and right circularly polarised light under unpolarised photoexcitation conditions. The CPL efficiency, |*g*_CPL_|, of BPP in the KBr pellet is of the order of 10^−3^, while the |*g*_CPL_| of BPP is ∼2.0 × 10^−3^ in the KBr-dispersed state.

**Fig. 5:**
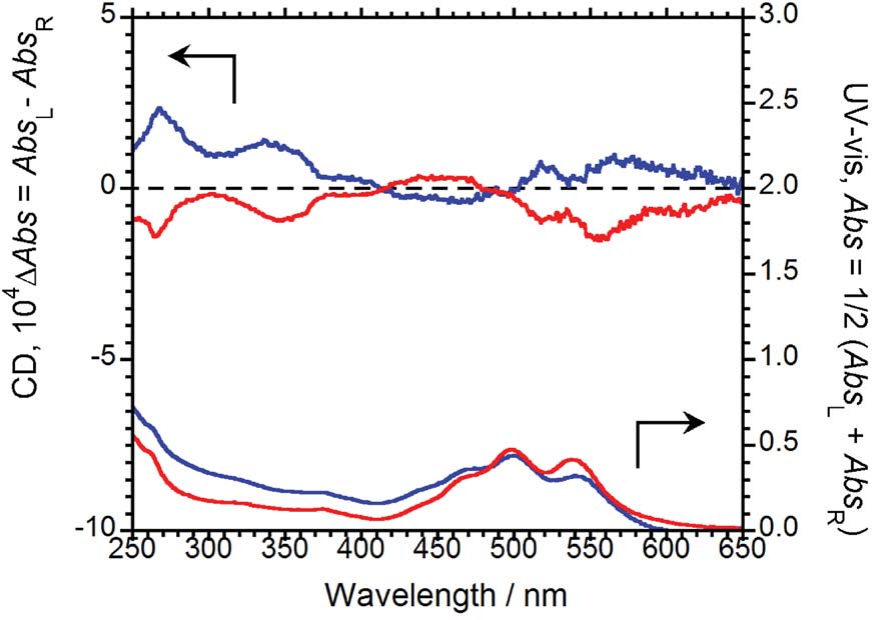
CD (upper) and UV-Vis (lower) absorption spectra of (*R*,*R*)-BPP (blue) and (*S*,*S*)-BPP (red) in KBr-pellet-dispersed states.

To examine molecular chirality in the ground state, the CD and UV-Vis absorption spectra of (*R*,*R*)-BPP and (*S*,*S*)-BPP in their KBr-pellet-dispersed states were acquired, the results of which are shown in Fig. 5.

Several UV-Vis bands between 400 and 650 nm, which are characteristic of π–π* transitions in the perylene groups of BPP, can be observed. In this state, the Cotton CD bands of (*R*,*R*)-BPP and (*S*,*S*)-BPP, are almost mirror images and are due to π–π* transitions, as evidenced by their UV-Vis spectra. The CD spectra of (*R*,*R*)-BPP and (*S*,*S*)-BPP are almost unaffected by KBr-pellet dispersion. Interestingly, the CD spectra of the KBr-dispersed states are quite different to those of the CHCl_3_ solutions are similar to those of the previously reported nanoscale aggregates in the solution-dispersed state.^4*g*^ We conclude that the CD spectra of the KBr-dispersed states also result from intermolecular interactions between the perylene units of multiple aggregated BPP molecules within the KBr pellets. To quantitatively evaluate the CD amplitude in the ground state, we evaluated the anisotropy factor. The magnitude of the circular polarisation in the ground state is defined as *g*_CD_ = (Abs_L_ − Abs_R_)/[(Abs_L_ + Abs_R_)/2], where Abs_L_ and Abs_R_ are the absorbances of the left and right circularly polarised light, respectively. The value of |*g*_CD_| at the first Cotton CD band of BPP in KBr was determined to be ∼5.4 × 10^−4^ at 561 nm. The observed CD results in KBr support the CPL observations mentioned above. Interestingly, a comparison of these anisotropy factors, *i.e.*, the |*g*_CPL_| and |*g*_CD_| values, reveals that the |*g*_CPL_| is larger than the corresponding |*g*_CD_| value, indicating that, although the perylene units are stacked and twisted in the ground state,^4*f*^ the twists increase in the photoexcited state, as confirmed by the intense excimer CPL bands.

**Fig. 6:**
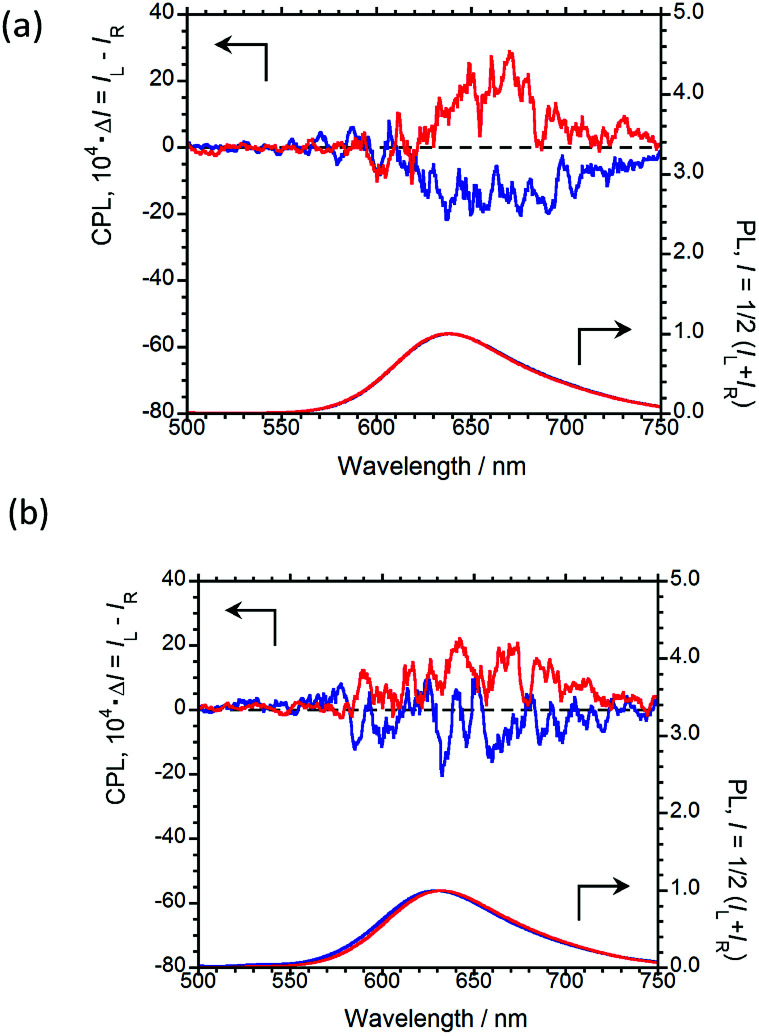
CPL (upper) and PL (lower) spectra of (*R*,*R*)-BPP (blue) and (*S*,*S*)-BPP (red) in (a) PMMA-film- and (b) *myo*-IPU-film-dispersed states.

In order to emit stronger CPL, we next examined CPL from organic polymer-film states. As an organic polymer matrix, poly(methyl methacrylate) (PMMA) and polyurethane (*myo*-IPU) derived from naturally occurring *myo*-inositol were used. The CPL and PL spectra of (*R*,*R*)-BPP and (*S*,*S*)-BPP dispersed in PMMA and *myo*-IPU films are shown in Fig. 6(a) and (b), respectively.

(*R*,*R*)-BPP in PMMA emitted at 654 nm (*λ*_CPL_) with a *Φ*_F_ of 0.40, while in *myo*-IPU, it emitted at 637 nm (*λ*_CPL_) with a *Φ*_F_ of 0.43. As expected, (*R*,*R*)-BPP exhibits solid-state AIEnh-CPL in both polymers, with several regressional phonon sidebands originating from the lowest π–π* transitions between perylene groups observed. The CPL spectra of (*R*,*R*)-BPP in PMMA and *myo*-IPU films mostly exhibited negative (−) CPL bands (Fig. 6(a) and (b), respectively). As expected, (*S*,*S*)-BPP exhibited positive (+) CPL spectra in these polymers (Fig. 6(a) and (b)). Although the CPL spectral shapes of (*R*,*R*)-BPP and (*S*,*S*)-BPP in the KBr-pellet and PMMA- and *myo*-IPU-film states are nearly mirror images, subtle differences in the shapes of the CPL signals are noted. These differences may be due to the effect of humidity in the pellets and films during the preparation of the pellet and film samples. The |*g*_CPL_| values of BPP in the PMMA and *myo*-IPU states are ∼2.4 × 10^−3^ and ∼1.3 × 10^−3^, respectively, which are similar to that observed in the KBr state (∼2.0 × 10^−3^).

As expected, despite the CPL wavelengths of (*R*,*R*)-BPP in the KBr and polymer states being similar, the values of *Φ*_F_ in these polymers are significantly higher than that observed for the KBr state (0.09). We assume that these approximately five-times-higher values are due to the suppression of thermal deactivation modes and effective packing arrangements because the molecular geometry of the chiral BPP is effectively and suitably fixed by the surrounding solid polymer matrix without quenching.

**Fig. 7:**
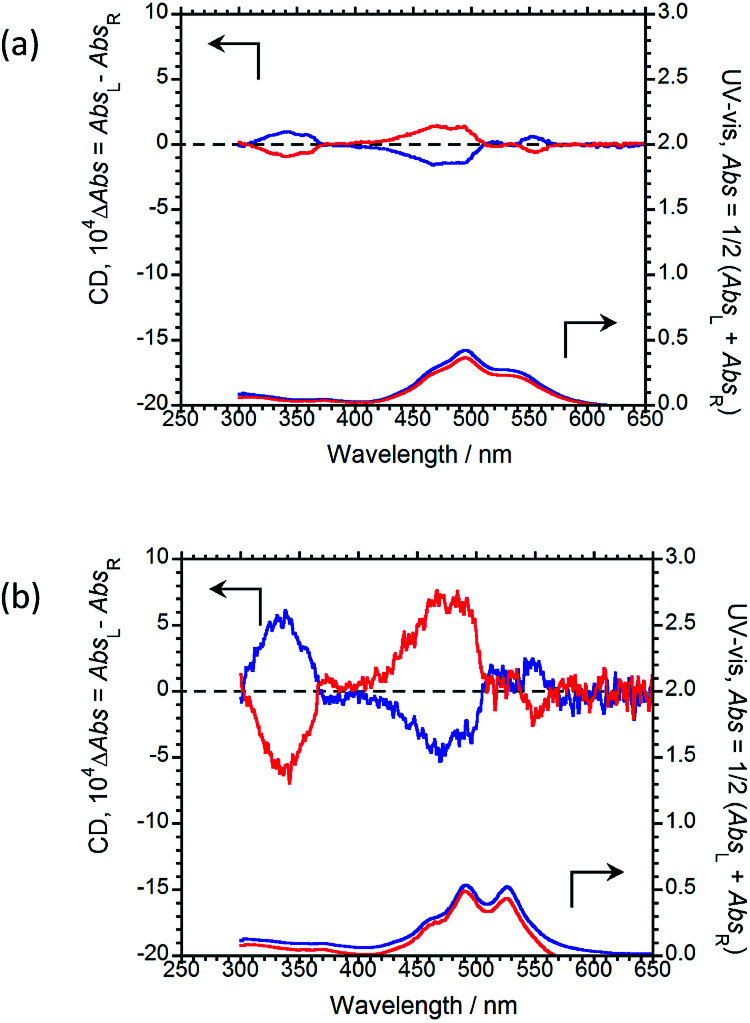
CD (upper) and UV-Vis absorption (lower) spectra of (*R*,*R*)-BPP (blue) and (*S*,*S*)-BPP (red) in (a) PMMA-film- and (b) *myo*-IPU-film-dispersed states.

The CD and UV-Vis absorption spectra of (*R*,*R*)-BPP and (*S*,*S*)-BPP in the PMMA and *myo*-IPU films are shown in Fig. 7(a) and (b), respectively; the CD spectra in these polymers are similar in their long-wavelength tails and are similar to those of the KBr-dispersed states. These results suggest that the CD spectra of the polymer-dispersed states are essentially due to intermolecular interactions between multiple molecules rather than intramolecular interactions within individual BPP molecules. The characteristic UV peaks originating from π–π* transitions of the perylene groups are commonly observed between 400 and 600 nm. The CD spectra of (*R*,*R*)-BPP and (*S*,*S*)-BPP in both matrices are almost mirror images, as expected. The signs of the first Cotton CD bands of (*R*,*R*)-BPP, at 553 nm in PMMA and at 551 nm in *myo*-IPU, are both positive (+), while the CD signs of the corresponding bands for (*S*,*S*)-BPP in PMMS and *myo*-IPU are both negative (−). The |*g*_CD_| value at the first Cotton CD bands are ~3.1 × 10^−4^ and ~2.9 × 10^−4^ for the PMMA- and *myo*-IPU-dispersed states, respectively. BPP in these matrices exhibit |*g*_CD_|values of similar magnitude.

As was observed in the KBr matrix, the |*g*_CPL_| values are higher than the corresponding |*g*_CD_| values in the polymer films. This shows that intermolecular twists between perylene units in the ground state increase upon photo-excitation, as evidenced by the intense excimer CPL bands in spectra of the polymer-matrix imbedded samples.

## Conclusions

Chiral (*R*,*R*)- and (*S*,*S*)-*N*,*N*′-bis(1-phenylethyl)perylene-3,4,9,10-tetracarboxylic diimide (BPP) dispersed in solid KBr and PMMA (or *myo*-IPU) matrices exhibited solid-state AIEnh-CPL with high *Φ*_F_ values at room temperature. The value of *Φ*_F_ from the PMMA (*Φ*_F_ 0.40) and *myo*-IPU matrices (*Φ*_F_ 0.43) are significantly higher than that observed from the KBr matrix (*Φ*_F_ 0.09). The present knowledge provides guidance for the design of novel solid chiral organic fluorophoric systems that emit solid-state AIEnh-CPL.

## Conflicts of interest

There are no conflicts to declare.

## Supplementary Material

RA-009-C8RA09785B-s001
