# Supplementary material for: Solid-state AIEnh-circularly polarised luminescence of chiral perylene diimide fluorophores
Source: RSC Adv. 2019 Jan 14;9(4):1976–81. doi: 10.1039/c8ra09785b (PMC9059715; doi:10.1039/c8ra09785b)
Supplement: RA-009-C8RA09785B-s001 [file RA-009-C8RA09785B-s001.pdf]

Electronic Supplementary Material (ESI) for RSC Advances.
This journal is © The Royal Society of Chemistry 2019

Electronic Supporting Information for

Solid-State AIEnh-Circularly Polarised Luminescence of Chiral Perylene Diimide Fluorophores

Ayano Taniguchi,[a] Daiki Kaji,[a] Nobuyuki Hara,[a] Ryosuke Murata,[b] Shogo Akiyama,[a] Takunori Harada,[c] Atsushi Sudo,[a] Hiroyuki Nishikawa,*[b] and Yoshitane Imai*[a]

[a] Department of Applied Chemistry, Faculty of Science and Engineering, Kindai University, 3-4-1 Kowakae, Higashi-Osaka, Osaka 577-8502, Japan. E-mail: (Y.I.) y-imai@apch.kindai.ac.jp.

[b] Graduate School of Science and Engineering, Ibaraki University, 2-1-1 Bunkyo, Mito, Ibaraki 310-8512, Japan. E-mail: (H.N.) hiroyuki.nishikawa.sci@vc.ibaraki.ac.jp

[c] Department of Integrated Science and Technology, Faculty of Science and Technology, Oita University, Dannoharu, 700, Oita city 870-1192, Japan

Table of Contents

Fig. S1 ^{1}H-NMR spectrum of (*R,R*)-**BPP** in $CDCl_3$ (500 MHz) S2

Fig. S2 ^{13}C-NMR spectrum of (*R,R*)-**BPP** in $CDCl_3$ (125 MHz) S2

Fig. S3 ^{1}H-NMR spectrum of (*S,S*)-**BPP** in $CDCl_3$ (500 MHz) S3

Fig. S4 ^{13}C-NMR spectrum of (*S,S*)-**BPP** in $CDCl_3$ (125 MHz) S3

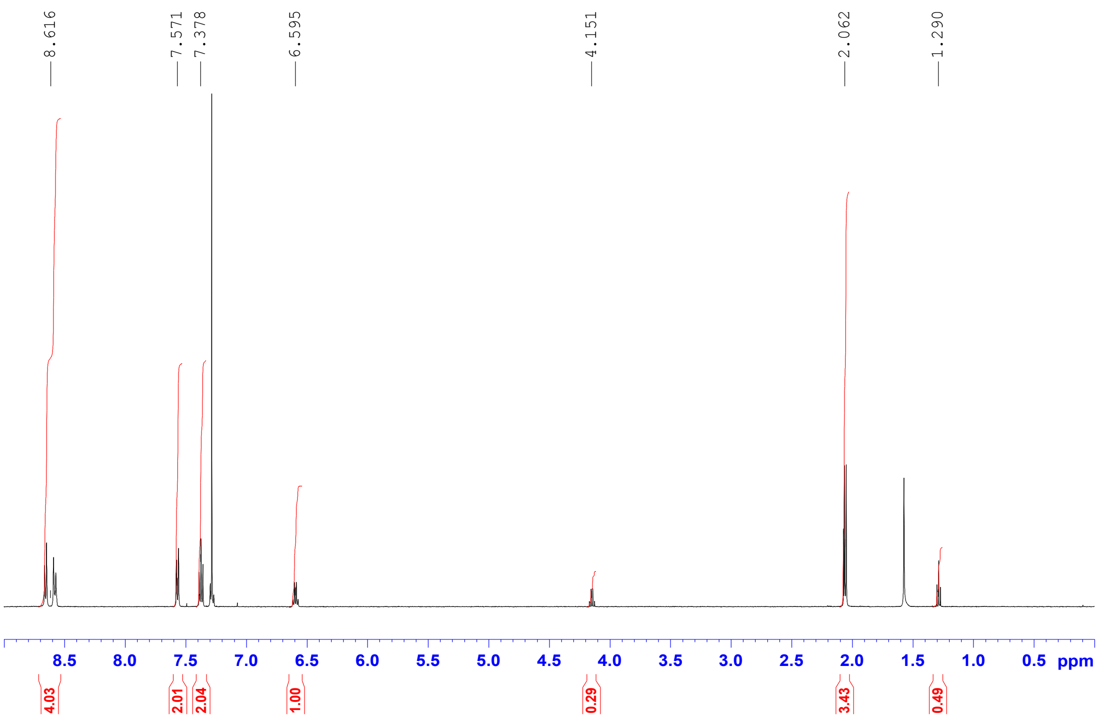

Fig. S1 ^{1}H-NMR spectrum of (*R,R*)-**BPP** in $CDCl_3$ (500 MHz).

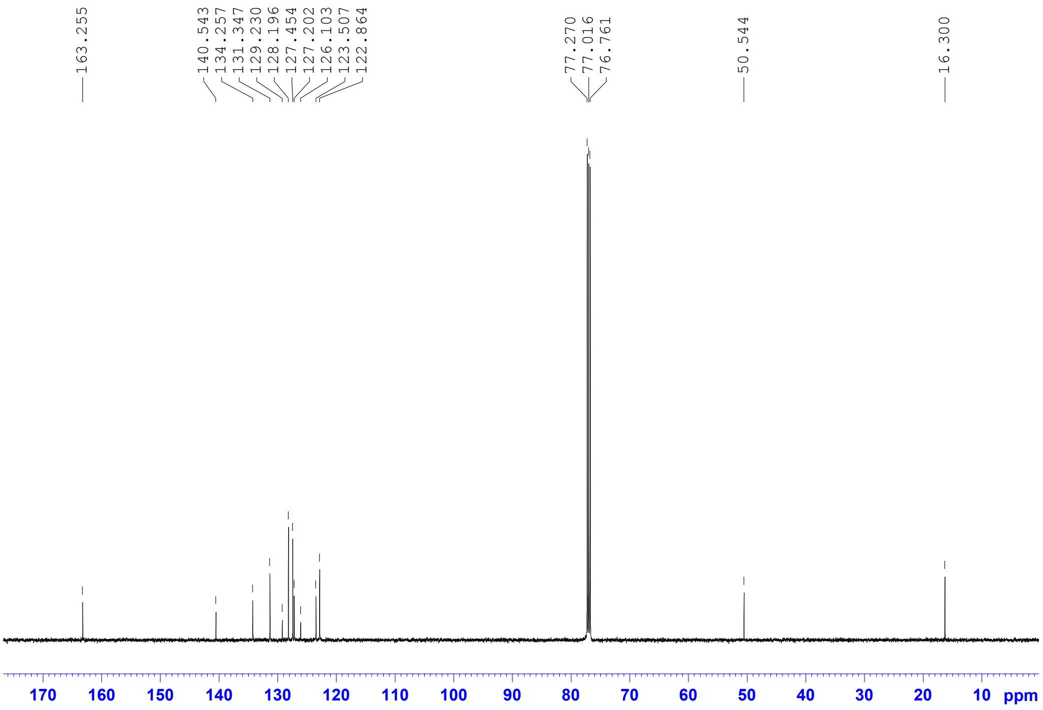

Fig. S2 ^{13}C-NMR spectrum of (*R,R*)-**BPP** in $CDCl_3$ (125 MHz).

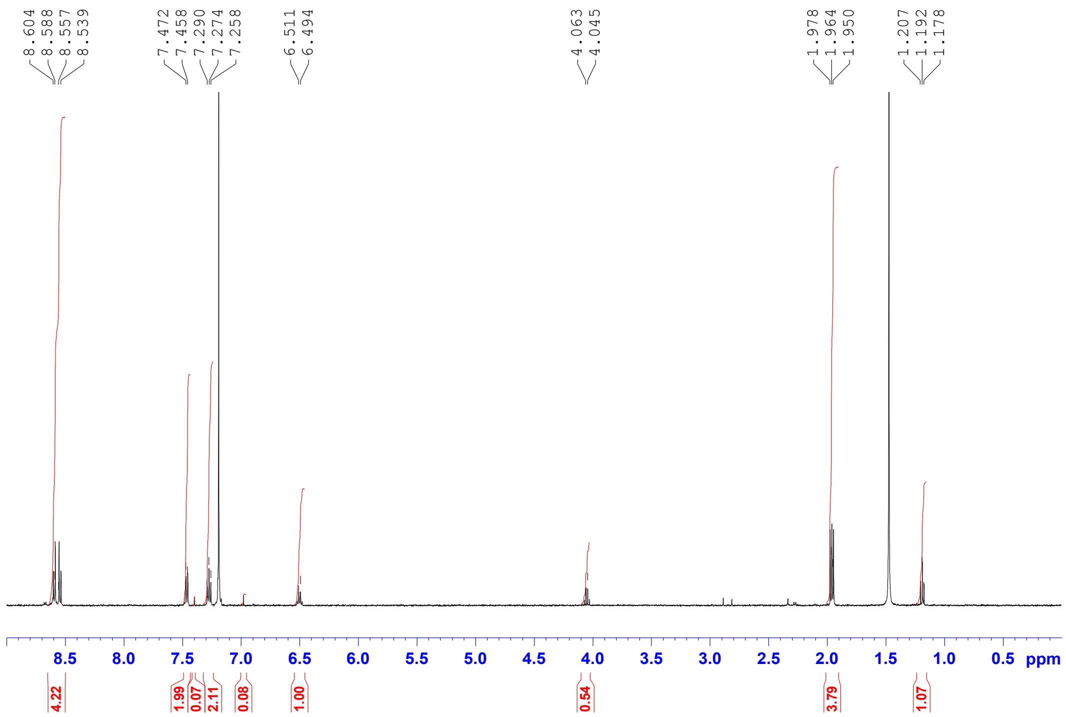

Fig. S3 ^{1}H-NMR spectrum of (*S*,*S*)-**BPP** in $CDCl_3$ (500 MHz).

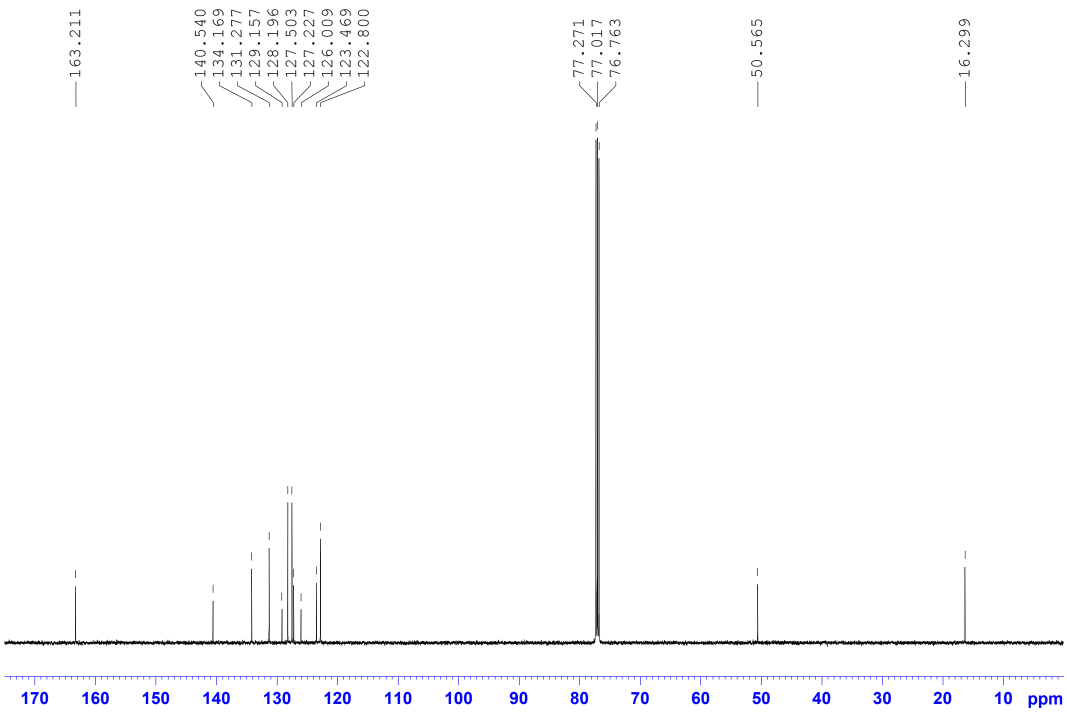

Fig. S4 ^{13}C-NMR spectrum of (*S*,*S*)-**BPP** in $CDCl_3$ (125 MHz).
